# Retraction Note: GDF11 enhances therapeutic functions of mesenchymal stem cells for angiogenesis

**DOI:** 10.1186/s13287-023-03247-1

**Published:** 2023-01-31

**Authors:** Chi Zhang, Yinuo Lin, Ke Zhang, Luyang Meng, Xinyang Hu, Jinghai Chen, Wei Zhu, Hong Yu

**Affiliations:** 1 Department of CardiologySecond Affiliated Hospital, School of Medicine, Zhejiang University, 88 Jiefang Rd, Hangzhou, 310009 Zhejiang Province People’s Republic of China; 2 Department of Obstetrics, Women’s Hospital, School of Medicine, Zhejiang University, Hangzhou, 310006 Zhejiang Province People’s Republic of China; 3 Department of Vascular Surgery, Hangzhou Third People’s Hospital, Hangzhou, 310009 Zhejiang Province People’s Republic of China; 4 Cardiovascular Key Laboratory of Zhejiang Province, 88 Jiefang Rd, Hangzhou, 310009 Zhejiang Province People’s Republic of China

**Stem Cell Res Ther (2021) 12:456** https://doi.org/10.1186/s13287-021-02519-y

The authors have retracted this article. After publication, the authors noticed image duplication errors in Figs. 2A, 5K, 6B, S3E and S10B, some of which also overlapped with the authors' earlier article [1]. Additionally, a wrong western blot image was used for Bcl-2 in Fig. S6G, and the flow cytometry plots in Fig. S7B were gated incorrectly. Due to the extent of these errors, the authors no longer have confidence in the presented data.

All authors agree to this retraction.
